# The Protein Kinase KIS Impacts Gene Expression during Development and Fear Conditioning in Adult Mice

**DOI:** 10.1371/journal.pone.0043946

**Published:** 2012-08-24

**Authors:** Valérie Manceau, Elisabeth Kremmer, Elizabeth G. Nabel, Alexandre Maucuer

**Affiliations:** 1 INSERM, UMR-S 839, Paris, France; 2 Université Pierre et Marie Curie, Paris, France; 3 Institut du Fer à Moulin, Paris, France; 4 Institute of Molecular Immunology, Helmholtz Zentrum München, München, Germany; 5 Brigham and Women's Hospital and Harvard Medical School, Boston, Massachusetts, United States of America; Florida State University, United States of America

## Abstract

The brain-enriched protein kinase KIS (product of the gene UHMK1) has been shown to phosphorylate the human splicing factor SF1 *in vitro*. This phosphorylation in turn favors the formation of a U2AF^65^-SF1-RNA complex which occurs at the 3′ end of introns at an early stage of spliceosome assembly. Here, we analyzed the effects of KIS knockout on mouse SF1 phosphorylation, physiology, adult behavior, and gene expression in the neonate brain. We found SF1 isoforms are differently expressed in KIS-ko mouse brains and fibroblasts. Re-expression of KIS in fibroblasts restores a wild type distribution of SF1 isoforms, confirming the link between KIS and SF1. Microarray analysis of transcripts in the neonate brain revealed a subtle down-regulation of brain specific genes including cys-loop ligand-gated ion channels and metabolic enzymes. Q-PCR analyses confirmed these defects and point to an increase of pre-mRNA over mRNA ratios, likely due to changes in splicing efficiency. While performing similarly in prepulse inhibition and most other behavioral tests, KIS-ko mice differ in spontaneous activity and contextual fear conditioning. This difference suggests that disregulation of gene expression due to KIS inactivation affects specific brain functions.

## Introduction

Gene expression is subject to a host of regulatory mechanisms, expanding the way in which cells can regulate their protein composition and setting the bases for the complexity of morphology and connectivity of neuronal cells. In addition to control at the level of gene transcription, the steps of pre-mRNA splicing, trafficking and translation in the gene expression process also are exquisitely regulated [1]. It is well established that phosphorylation regulates transcription factors, but little is known about its role in the regulation of splicing factors [2].

The splicing factor SF1 is phosphorylated *in vivo* on two serines within a highly conserved SPSP motif and substrates for the protein kinase KIS *in vitro* [3]. At an early step of spliceosome assembly SF1 interacts with the branch point sequence and the 65 kDa subunit of U2AF (U2AF^65^), which binds the nearby polypyrimidine tract [4]–[10]. We previously observed that the dual phosphorylation of SF1 on its SPSP motif enhances SF1 interaction with U2AF^65^ [3]. In addition, using an intronic acceptor RNA substrate, we showed that phosphorylation of SF1 favors the formation of a ternary RNA-U2AF^65^-SF1 complex. This suggested a function of SF1 phosphorylation by KIS in the recognition of the 3′ splice site. Additional functions have been proposed for KIS, including the phosphorylation of p27kip1, stathmin and PAM [11]–[14].

In mammals, KIS mRNA is expressed ubiquitously with a particular enrichment in the nervous system [13], [15]. A function of KIS in smooth muscle cell migration has been documented using a model of atherosclerosis in KIS-deficient (KIS-ko) mice [14]. During postnatal development of the mouse brain, the KIS mRNA level increases gradually, reaching its highest level in the mature brain [15]. Experiments using primary cultures of cortical mouse neurons indicated a function for KIS in neuritic elongation and mRNA translation in dendrites [16]. However, no study has directly tested the role of KIS in brain development and functions. Here, we assess the effect KIS has on neural development using KIS-deficient (KIS-ko) mice. We show that KIS is necessary for normal phosphorylation of SF1 *in vivo* and regulates the expression of sets of genes in the neonate brain. While surprisingly KIS deletion does not induce significant alteration in neuritic elongation of neurons in culture, we demonstrate that KIS is important for normal spontaneous activity and fear conditioning of adult animals, suggesting a more subtle function for KIS in brain programming during development.

## Results

### Coexpression of KIS and SF1 in mouse tissues and during brain development

**Figure 1:**
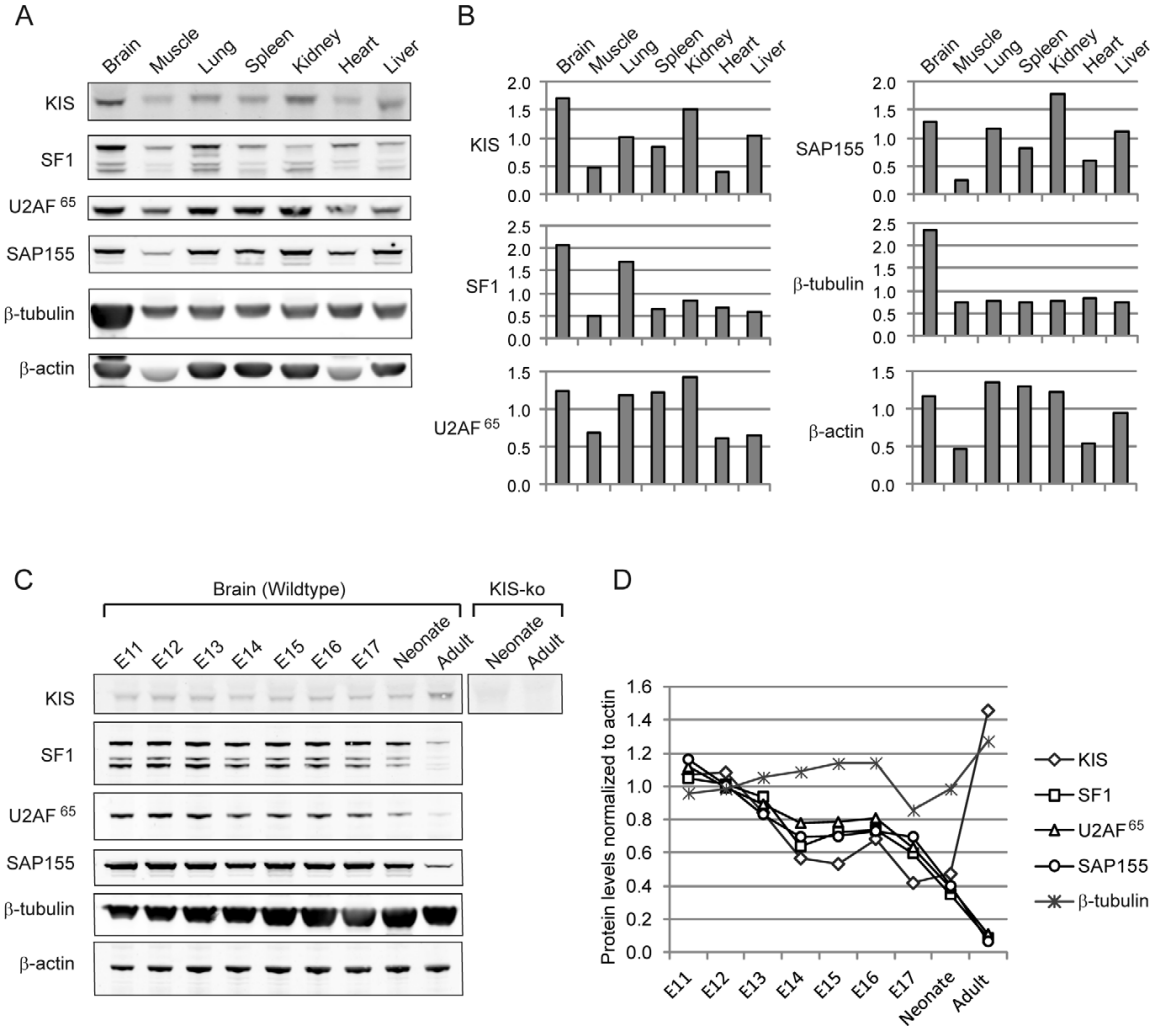
Expression of KIS, SF1 and related splicing factors in different mouse tissues and during brain development. A. Expression in different neonate tissues. 10 µg of total proteins were loaded on 10% acrylamide SDS PAGE and analyzed by Western blot using different antibodies. B. Western blots quantification. Expression of KIS and SF1 were ubiquitous but higher in brain. As expected β-tubulin levels were higher in brain and β-actin was lower in skeletal muscle and heart [41]. C. Expression in brain during development. The profile of expression of proteins during brain development was assessed by western blot. Brains extracts from neonate and adult brains of KIS-ko animals were used to control the specificity of the anti KIS antibodies (right panel). D. Western blot quantification. The signal for the different proteins was normalized to β-actin and to the mean of E11 to E13 signals. Similar profiles were observed for splicing factors SF1 and U2AF^65^ which are known to functionally interact [7] and for the U2snRNP component SAP155 that interacts with U2AF^65^ and KIS [25], [42].

To assess the physiological significance of the interaction of KIS with SF1, we first analyzed the tissue distribution of these two proteins (figure 1A and 1B). In the neonate mice, both KIS and SF1 showed the highest expression in the brain as compared to the other tested tissues, in agreement with previous expression analyses [12]–[14], [17], [18].

We next analyzed the expression of KIS and of the splicing factors SF1, U2AF^65^ and SAP155 during brain development (figure 1C and 1D). The specificity of the 47 kDa band detected with the monoclonal anti-KIS antibody was confirmed using tissues from KIS-ko animals. All four proteins presented a similar pattern of expression during embryonic development. However KIS level was increased in the adult brain in contrast to the splicing factors whose levels are about tenfold less in adult compared to embryonic day 12. These expression profiles support the notion that KIS and SF1 might interact in various tissues, particularly in the developing brain.

### KIS knock-out affects migration of SF1 in polyacrylamide gel electrophoresis

**Figure 2:**
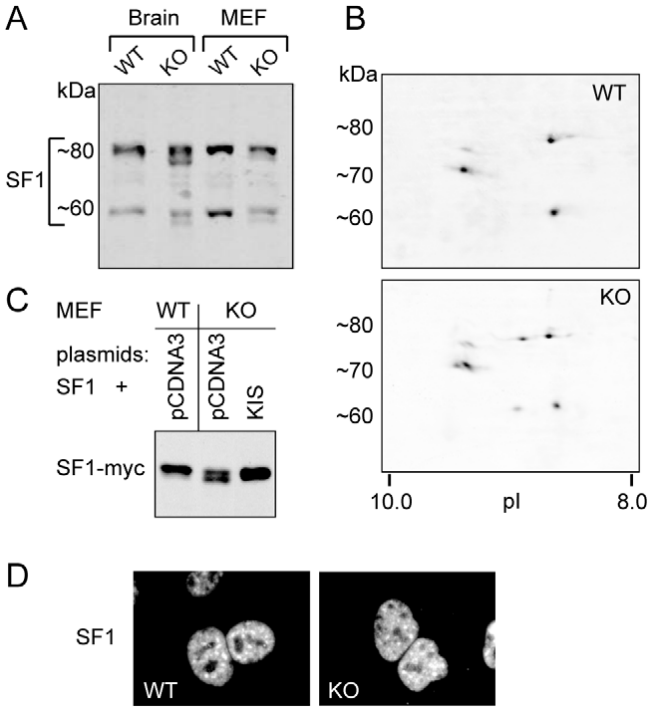
SF1 expression in KIS-ko mice. A. Total protein extracts from neonate brain and from embryonic fibroblasts from wildtype and KIS-ko mice were analyzed by Western blot. B. Protein extracts from brain of neonate mice were analyzed by 2D electrophoresis and western blot. C. SF1 with a C-terminal myc tag was expressed in MEF cells derived from wild type or KIS-ko mice. Cells were cotransfected for KIS expression or with control vector (pCDNA3). D. MEF cells were processed for immunofluorescence with anti-SF1 antibody and observed with a x40 objective.

We next looked for consequences of KIS knock-out on SF1 expression in the brain of neonate mice as both KIS and SF1 were expressed at significant levels at this stage. On immunoblots SF1 appears as multiple bands due to alternative splicing of its pre-mRNA [3], [18] (figure 2A, lanes 1 and 3). We have previously shown that SF1 is extensively phosphorylated in brain and HEK293 cell extract since its dephosphorylation in these extracts leads to the appearance of additional faster migrating SF1 isoforms in polyacrylamide gel electrophoresis [3]. Comparing brain and fibroblast extracts in wild type and KIS-ko mice we similarly observed an additional faster migrating band for each of the major SF1 bands when KIS was absent (figure 2A). Therefore KIS deletion apparently had the same effect as partial dephosphorylation on SF1 migration. We further analyzed these isoforms using two-dimensional gel electrophoresis (figure 2B). More basic species for each of the SF1 spliced isoforms were detected in KIS-ko extracts, in agreement with the hypothesis that SF1 is partially unphosphorylated when KIS is absent. Quantification of the different signals on 1D and 2D blots indicated that the total level of SF1 protein was unchanged but that about 30% of SF1 had a modified migration. We then expressed KIS together with myc-tagged SF1 in MEF cells derived from wildtype or KIS-ko animals. KIS re-expression restored a wild type pattern with a unique SF1-myc band (figure 2C). Therefore KIS deletion changed the pattern of migration of SF1 isoforms, most probably because of defects in SF1 phosphorylation. In contrast the nuclear distribution of SF1 was apparently not affected by the deletion of KIS (figure 2D).

### KIS knock-out affects gene expression in neonate brain

**Figure 3:**
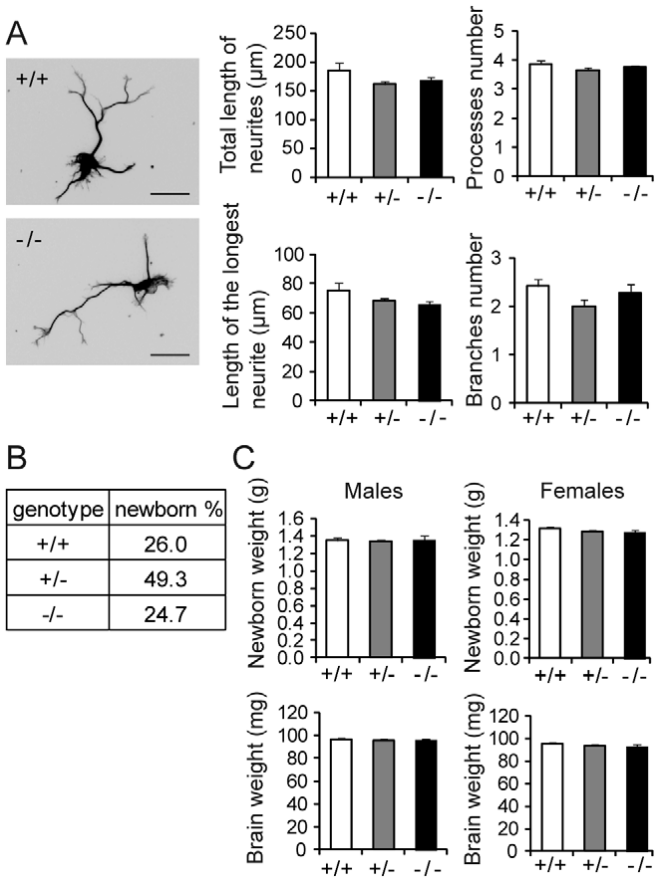
Analyses of neuritic extension of embryonic cortical neurons, embryonic viability and newborn weight. A. Neuritic extension of cortical neurons from E14 embryos was analyzed after four days in culture by immunolabelling with anti-actetylated tubulin antibodies (bar: 20 µm). For each culture a minimum of 150 neurons were analyzed. Mean values for the total length of the neurites, the length of the longest neurite, the number of processes and the number of branches are presented. Error bars are standard errors of the mean. B. The genotype of 146 newborns from heterozygous matings was determined and genotype frequencies determined. C. The weight of neonate and of their brain was determined for 82 females and 64 males. The mean values for each genotype are presented.

The expression of KIS and SF1 being highest in the brain, we addressed the potential functional consequences of KIS deletion in the developing brain. First, KIS-ko mice were backcrossed for 10 generations to get a homogenous C57BL6/N genetic background. It was reported that KIS knockdown using shRNAs has a dramatic effect on neuritic elongation of embryonic cortical neurons in culture [16]. Surprisingly, comparison of similar primary cultures derived from +/+, +/− and −/− E14 embryos did not reveal a significant difference in neuritic arborization of cortical neurons (figure 3A). KIS-ko newborns were indistinguishable from their wild type siblings and inheritance ratios of the mutated allele indicated no effect of KIS deletion on embryonic viability (figure 3B). No differences in total weight or brain weights of KIS-ko newborns could be detected (figure 3C). Altogether KIS deletion had no apparent effect on embryonic viability, brain weight, or neuritic elongation of cortical neurons when cultured *in vitro*.

**Figure 4:**
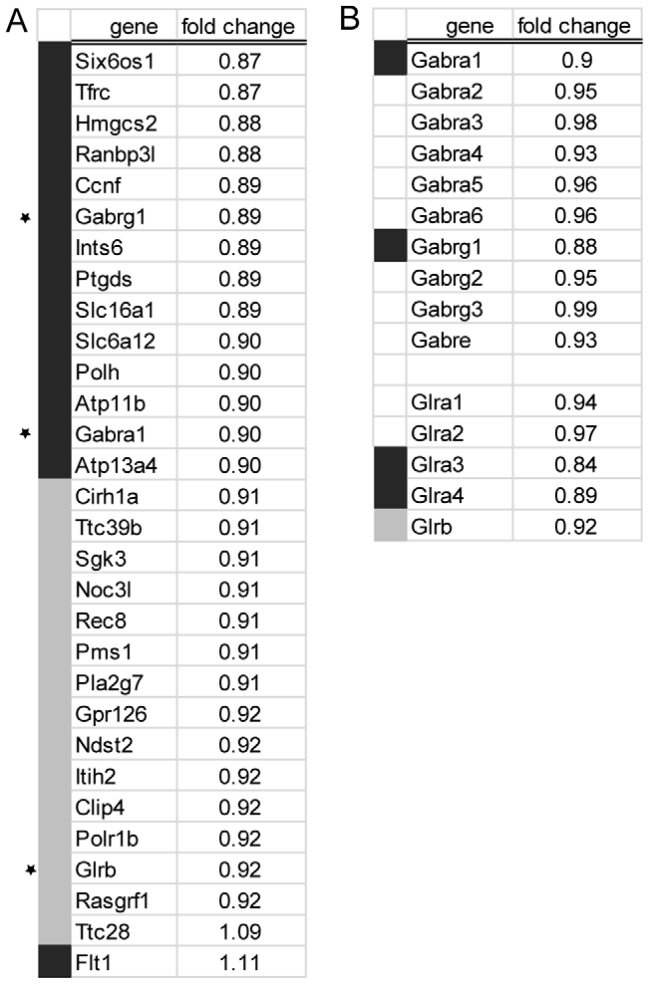
Microarray analysis of gene expression in KIS-ko brain. A. Total RNA from the brains of six pairs of wildtype and KIS-ko neonate littermates was processed for Exon 1.0 ST array hybridization (Affymetrix). Genes with fold changes above 6% were ranked according to the confidence values. The 80 top genes were then ranked for the difference value and the top 30 of this list are presented with a color code for fold change (dark grey>10%, light grey>8%). Stars indicate cys-loop ligand-gated channels in this list. B. GABA_A_ and glycine channel receptor subunit genes showing expression changes in the microarray dataset are listed.

**Figure 5:**
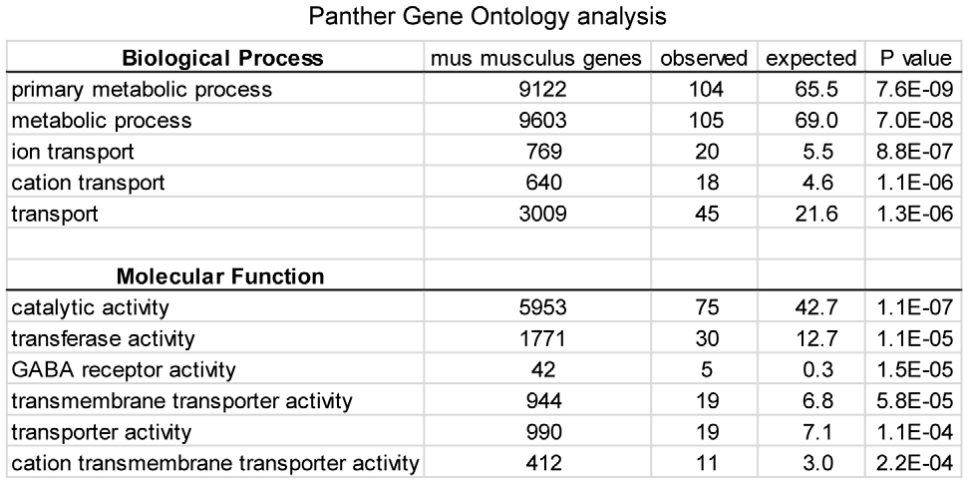
Gene ontology analysis of genes differently expressed in the brain of adult KIS-ko animals. Analysis was performed on the top 200 genes differently expressed in KIS-ko mice according to the confidence values, using the Panther Gene Ontology gene annotation package.

Given the putative function of KIS in gene expression regulations, we next compared the transcriptome of KIS-ko neonate brains with that of +/+ littermates using a microarray approach. Six pairs of brains from +/+ and −/− neonate littermates were used for probing mouse exon 1.0 ST arrays from Affymetrix. These arrays provide an accurate measure of transcript levels, since four probes are present for each known or predicted exon in the transcriptome. In addition, prominent splicing changes can be detected by searching for differences in the splicing indexes. Although such splicing changes were not detected in the KIS-ko neonate brains, we repeatedly noticed genes with a reduced expression of all exons. We therefore applied a measure of gene expression based on the detection level of all the probesets for each gene (see Material and Methods). Among 16,500 genes for which expression could be quantified, 20% had a change in expression level of at least 6%. For each gene a confidence value for expression difference was scored. The 30 genes with the highest differences among the 80 genes with the highest confidence are listed in figure 4A. 28 of them presented a downregulation in KIS-ko brains and surprisingly three of these genes corresponded to subunits of cys-loop ligand-gated ion channels (Gabrg1, Gabra1 and Glrb). Using the list of the 200 genes with the most significant difference in expression and the Panther software package for gene ontology analysis [19] we confirmed the significant enrichment of genes related to ion transport and metabolic processes (figure 5). Further analysis of the microarray dataset revealed that other GABA_A_ and glycine receptor subunits were also potentially down-regulated (figure 4B).

**Figure 6:**
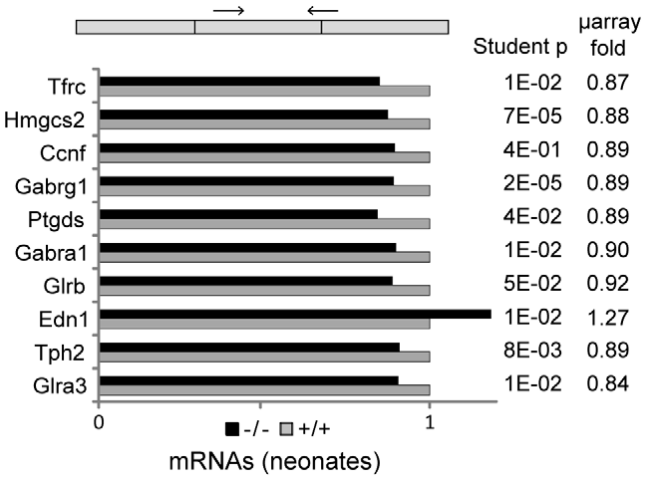
Q-PCR analyses of mRNAs in brain of wildtype and KIS-ko neonate mice. Pairs of primers for mRNAs were chosen so that the PCR product crossed an exon-exon boundary. The mean values of mRNA levels relative to wildtype for 16 −/− and 16 wildtype littermates are presented. Paired student t-test values for the qRT-PCR data are indicated as well as the fold changes that were deduced from the microarrays analysis for comparison.

Q-PCR analyses using total RNA from the brains of 16 pairs of +/+ and −/− littermates confirmed the significant differences that were detected in the microarray analysis for nine out of ten genes (p<0.05, figure 6). In contrast we found no expression difference for the six of these genes that we tested in the adult (Tfrc, Hmgcs2, Ptgds, Gabra1, Tph2 and Glra3) (not shown).

**Figure 7:**
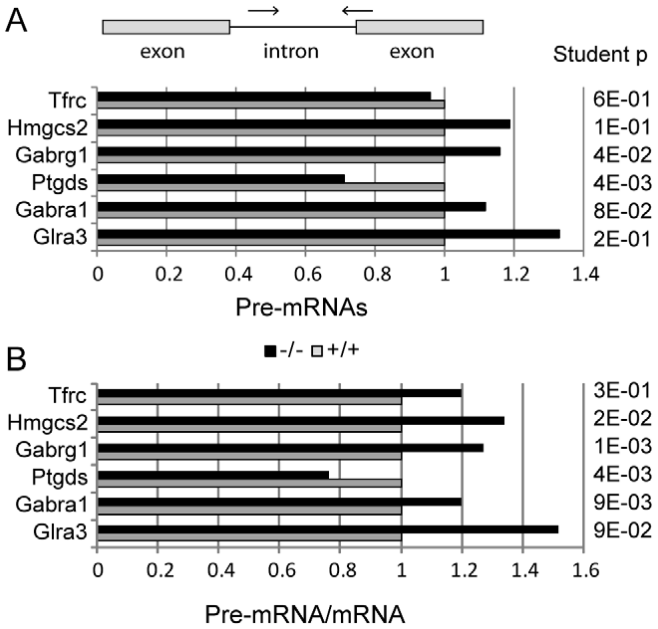
Q-PCR analyses of pre-mRNAs in brain of wildtype and KIS-ko neonate mice. A. Pairs of primers specific for pre-mRNAs were chosen with one primer in an intron and the other in an exon or crossing an intron-exon boundary. The mean values of pre-mRNA levels relative to wildtype for 16 −/− and 16 wildtype littermates are presented. B. For each gene the mean pre-mRNA over mRNA ratio is presented.

The lower levels of transcript that we observed in the neonate brain might result from lower transcription, altered rate of maturation of pre-mRNAs or increased degradation of mRNAs. To document these different hypotheses we further measured pre-mRNAs levels for some of the down-regulated genes (figure 7). Three out of six genes showed a significant increase in pre-mRNA/mRNA ratio in KIS-ko animals (Hmgcs2, Gabrg1 and Gabra1, p<0.05, figure 7B). These results suggested an alteration of the maturation of mRNA of the genes, the expression of which is decreased in KIS-ko mice.

### KIS deletion results in hyperactivity and altered fear learning

We next hypothesized that the cumulative effect of the moderate modifications in gene expression in KIS-ko neonate could result in defective brain functions in the adult. As no previous experiments had explored the consequence of KIS deficiency on animal behavior, we selected a variety of tests including memory and anxiety-related tests. In addition, given the reports of an association between schizophrenia and SNPs in the *UHMK1* gene (encoding the KIS protein) [20], [21] we searched for reduced prepulse inhibition, which is a known endophenotype for schizophrenia and is considered as a useful phenotype for detecting models of schizophrenia in mouse [22].

**Figure 8:**
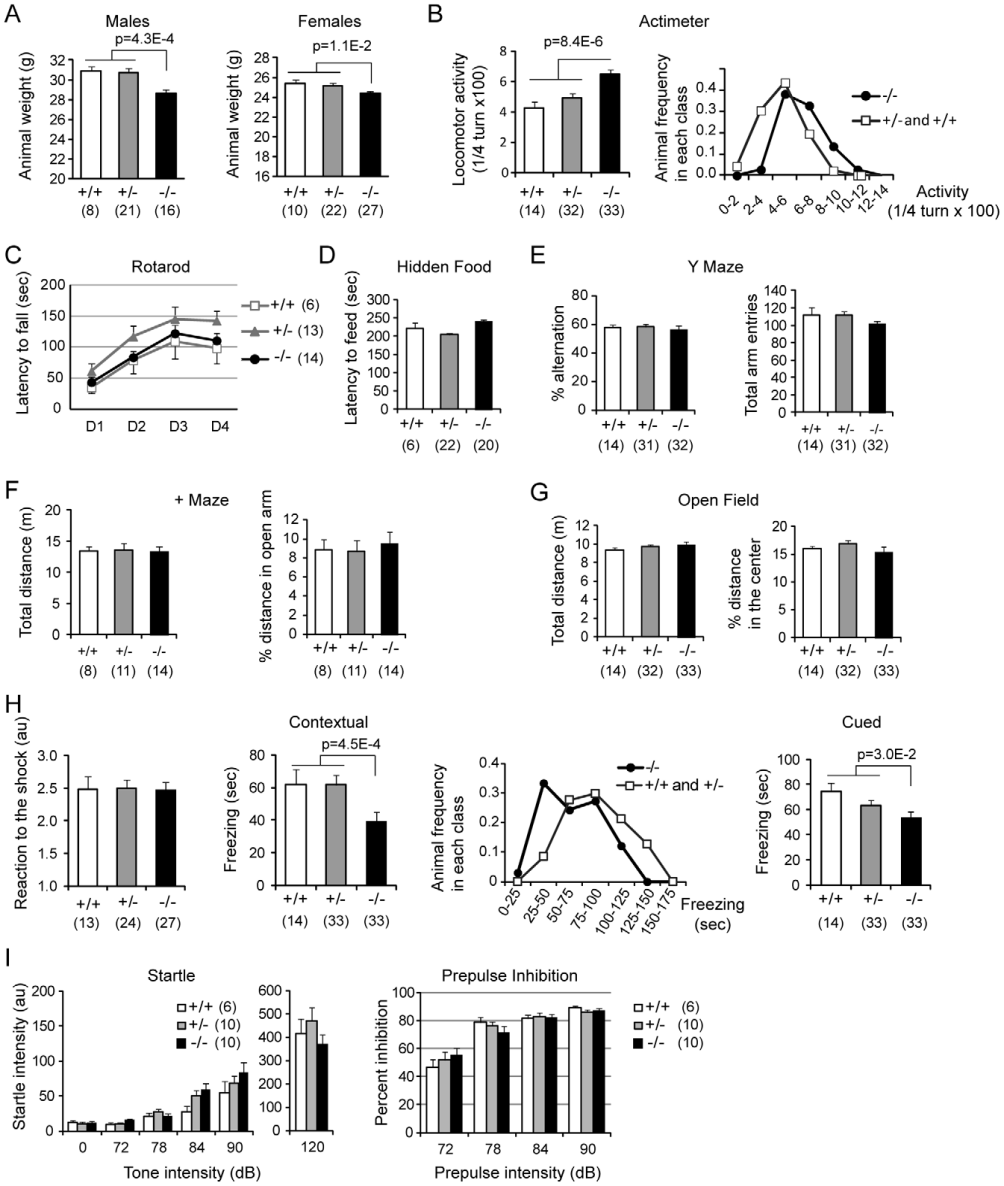
Analysis of KIS-ko mice physiology and behavior. A. Weights of adult animals of different genotypes. Numbers of animals are under brackets. B. Spontaneous activity of mice when placed in a circular corridor maze for two hours. A histogram for the repartition of the animals in the different classes for activity shows a bell shape justifying the ANOVA analysis. C. Performance of the animals in the rotarod test was evaluated for four consecutive days using acceleration from 4 to 40 rpm over five minutes. D. The animals' latency to find a hidden piece of raisin was calculated in a one session experiment. E. Y maze experiment: animal displacement was monitored in a 10 min session and the percentage of alternation was calculated. F. Animal anxiety was assessed in the plus maze test. The percentage of distance in the open arm is indicative of the anxiety of the animals. G. Similarly no difference in anxiety was revealed using the open field paradigm. H. Memory of context and cue assessed in a fear conditioning protocol. No difference in shock response was noticed between wild type and KIS-ko animals suggesting that pain sensitivity was not compromised (left panel). Measure of context memory was conducted 24 hours later by measuring freezing time in the conditioning environment (contextual). The histogram for the distribution of animals according to freezing time showed a bell shape indicating a homogenous population for each genotype. Two hours later animals were tested for freezing in a novel environment for three minutes without the tone (for background substraction), then for three minutes with the tone cue (cued). I. Startle response to increasing tone intensities show similar reactivity of the animals. A tone prepulse efficiently inhibited the startle response to the 120 dB tone, with no difference among genotypes.

No gross morphological or behavioral alterations were noticed when observing adult wild type and knockout animals in their home cage as previously observed in a mixed C57Bl/6-129SV genetic background [14]. In addition no particular mortality was observed. KIS-ko animals presented normal eye blink, forepaw reaching response as well as righting and postural reflexes. However there was a slight but significant decrease in the weight of the KIS-ko animals (figure 8A, 7% less body weight for males and 3% for females).

We then tested the spontaneous locomotor activity of the animals when placed in a dimly illuminated circular corridor commonly used to evaluate the response of animals to psychostimulant [23]. The KIS-ko animals were found to be significantly more active (figure 8B). No difference in any of our tests was observed between +/+ and +/− animals.

In the rotarod test, KIS-ko animals were indistinguishable from their wild type littermates, with similar initial performance and subsequent improvement over four consecutive days of testing, indicating normal basal motor skills and motor learning (figure 8C). No difference in olfactory perception was observed in a hidden food retrieving test (figure 8D). From this first series of tests, we could conclude that no major abnormality of vision, olfaction or motor skills was present in KIS-ko animals that could interfere with the anxiety related or memory tests that followed.

In the Y maze paradigm no significant difference in either alternation or total arm entries was observed indicating normal activity and spatial working memory (figure 8E). In the plus-maze and the open field tests, the total distance that was travelled by the animals was not significantly different between the genotypes (figure 8F and 8G). The exploration of aversive areas that are the open arms of plus maze and the center of open field were also similar in the two genotypes, suggesting no increase in anxiety behaviors in KIS-ko mice.

We next tested the memory of the animals using a fear conditioning protocol. On the training day, animals were placed in a novel context and after a 2 minutes exploration period, they were exposed to a tone in association with an electric foot shock. We observed no freezing of wild type and KIS-ko animals before the foot shock. There was also no difference in reaction to the foot shock between wildtype and KIS-ko animals (figure 8H, left panel). 24 hours later, KIS-ko animals presented a clearly reduced freezing response to the context and to a lesser extent to the tone cue (figure 8H).

Finally, we observed a similar startle response to an acute tone for KIS-ko and wild type animals suggesting no auditory alteration, and a similar inhibition of the startle response by a prepulse tone (figure 8I).

Altogether, although KIS-ko mice showed normal sensory responses, anxiety levels, and motor skills, their reduction in weight, their hyperactivity in a novel environment, and a distinct deficit in fear conditioning clearly distinguished them from the wild type.

## Discussion

### In vivo link between KIS and splicing factor SF1

The brain enriched protein kinase KIS presents a unique primary structure as it is formed by the juxtaposition of a protein kinase and a splicing factor related domain with a U2AF Homology Motif (UHM) [17], [24]. This unique organization first suggested KIS has a role in the phosphorylation of splicing factors [17]. UHMs, named for their initial identification in both subunits of splicing factor U2AF, bind peptide motifs known as UHM Ligand Motifs (ULM) with a high degree of specificity [24]. The UHM domain of KIS is necessary for its interaction with the ULM-containing splicing factor SF1 [3], [25]. In addition, KIS phosphorylates SF1 *in vitro* with a high efficiency on two residues that are highly phosphorylated *in vivo* [3], [25]. Here we observed that KIS and SF1 present a remarkable coexpression in the brain. However, both proteins were expressed in all the tissues examined, suggesting that the KIS-SF1 interaction occurs in all cell types. The profile of SF1 isoform migration on 1D and 2D SDS-PAGE is different for KIS-ko extracts in a way that strongly suggests an abnormal phosphorylation of SF1. This is the first observation of a biochemical consequence of KIS deletion in the animal and strong evidence that KIS directly acts on SF1 *in vivo*. However, it must be noted that in KIS-ko mice SF1 remained partly phosphorylated, indicating that at least one other kinase is responsible for SF1 phosphorylation in mice.

### Altered gene expression related to KIS deletion

KIS-ko neonate and adult mice did not present an obvious phenotype. KIS deletion had no apparent effect on embryonic viability and brain weight. We did not observe the dramatic effect of shRNA-mediated KIS knockdown on neuritic extension observed by Cambray et al. [16], which might instead be due to compensation in mice or off target effects of shRNAs.

However, the use of exon 1.0 ST arrays from Affymetrix allowed us to detect with a high confidence subtle transcript levels modifications that we then confirmed by Q-PCR. It is widely accepted that SF1 is implicated in early spliceosome assembly. Immunodepletion experiments of SF1 allowed to demonstrate a facilitating action for this factor on splicing, albeit not a strict requirement [26] as also supported by knockdown experiments in HeLa cells [27]. However SF1 function appears essential for cell life as its depletion in HeLa cells leads to cell death [27]. Similarly, SF1 knockout is lethal at embryonic stage in mice and required for *C. elegans* and yeast viability [6], [28]–[30]. Therefore, SF1 might be necessary for either achieving splicing kinetics required for cell life or for some essential splicing events. In KIS-ko mice, we could not detect splicing changes for distinctive exons within genes, but this could simply be due to the low sensitivity of exon arrays for measuring alternative splicing. In contrast we detected a global decrease of expression of all exons for a number of genes including subunits of the GABA_A_ and glycine receptors. Some of these subunits had a higher expression in the adult mice while others had a higher expression in the neonate (data not shown), indicating that the differences between wild type and KIS-ko mice were not due to a developmental delay. Although gene expression modifications were expected given the proposed function of KIS in RNA metabolism regulation, the precise mechanisms that are affected are now to be characterized. Genes for the subunits of the GABA_A_ and glycine receptor are clustered on a few chromosome loci and it has been proposed that such clustering would enable common expression regulations [31], [32]. However the subunits affected in KIS-ko animals were in different loci and not all subunit genes in one locus were affected. In addition, we did not observe any clustering of other affected genes. It is therefore unlikely that KIS affects gene expression through a mechanism of global control of clustered genes. Q-PCR analyses revealed an increase of pre-mRNA/mRNA ratio for Gabrg1, Gabra1 and Hmgcs2, suggesting that a splicing defect could be responsible for their reduced expression at the mRNA levels in agreement with a functional link between KIS and splicing factors including SF1 [3], [25]. As growing evidence indicate that splicing factors are normally actively recruited to transcription sites, it is possible that common sequence features or epigenetic determinants could make some genes more sensitive to reduced concentration of fully active SF1 and explain the specific requirement of KIS for their normal splicing [33]. However we cannot exclude other hypotheses. For example the increase in the pre-mRNA/mRNA ratio could result from a decreased stability of mRNAs potentially linked to an alteration of the proposed function of SF1 in RNA trafficking [34]. Further experiments are needed to address the direct involvement of SF1 in the processing of the transcripts that we found affected in KIS-ko brains. We also cannot exclude the possibility that the observed effects of KIS deletion partly result from altered phosphorylation of other proteins than SF1, or are indirect consequences of splicing defects of some pre-mRNAs. Further studies of KIS and SF1 will help answer these questions.

### Effect of KIS gene disruption on animal behavior

The higher expression of KIS in the nervous system and the modified expression of genes in the brain of KIS-ko animals suggested that KIS deletion could affect animal behavior. We performed a screening for behavioral defects of KIS-ko mice that allowed us to detect abnormalities associated with KIS deletion and to exclude defects in others as notably prepulse inhibition. KIS-ko mice displayed locomotor hyperactivity in the circular corridor novel environment. In contrast no hyperactivity was detected in the Y-maze, plus-maze or open field, suggesting that this context-dependent hyperactivity affects only locomotion and not the more exploratory behaviors. Second, we noticed a reduced contextual (and to a lesser extent cued) fear conditioning of KIS-ko animals, showing reduced learning capacities from aversive stimuli. It is unlikely that the reduced freezing resulted from hyperactivity as we found no inverse correlation between animal activity in the circular maze and freezing in the fear conditioning protocol. Furthermore our data obtained with large groups of animals yielded a very high statistical significance for the increased activity (Student p = 8.4E-6) and altered freezing in the contextual fear conditioning protocol (Student p = 4.5E–4) even after correction for the number of parameters (16) that have been measured along this behavioral screening (p = 1.3E–4 for increased activity and p = 7.2E–3 for altered contextual fear conditioning after Bonferroni correction). Our analysis provides therefore a sound basis for further experiments aiming at refining the behavioral characteristics of these mice and in particular their learning deficits. Interestingly we observed no difference in the rotarod test and normal alternation in the Y maze indicating that motor learning and spatial working memory are not affected by KIS deletion. Further analyses of KIS-ko mice learning ability in other memory related paradigms will be of interest to further refine their learning deficit.

Altogether our gene expression and behavioral analyses support a function of KIS in the fine tuning of gene expression during brain development that is important for adult brain functions. The significant enrichment of ion channels subunits among genes that are affected in the neonate brain suggests that a change in synaptic function could lead to deficits in the activity-dependent maturation of brain wiring and resulting abnormal behavior of adult animals. However our microarray and Q-PCR analyses indicated that KIS deletion affected a variety of genes including also metabolic enzymes as revealed by the gene ontology analysis (as Prostaglandin D2 synthase, 3-hydroxy-3-methylglutaryl-CoA synthase 2 and Tryptophan hydroxylase 2). Therefore the phenotype of KIS-ko mice might result from the cumulative effect of downregulations of a variety of genes.

### KIS and schizophrenia

Divergent results have been reported concerning the potential implication of KIS in the etiology of schizophrenia. While two reports established a genetic linkage with two SNPs in the KIS gene using independent British case-control samples [20], [21], such an association could not be reproduced in Bulgarian and French cohorts of patients [35], [36]. In addition KIS alterations have not been detected in individual samples [36], [37]. Interestingly, our present results support the possibility that KIS defects participate in perturbations of brain function associated with psychiatric diseases. The hyperactivity of animals is a potential model for positive symptoms in schizophrenic patients. However the normal prepulse inhibition in KIS-ko mice suggests that lack of KIS by itself does not provide a cause for this endophenotype in schizophrenia.

## Materials and Methods

### Ethics statement, Animals

Animal care was conducted in accordance with the standard ethical guidelines (National Institutes of Health publication number 85–23, revised 1985; European Community Guidelines on the Care and Use of Laboratory Animals; and French Agriculture and Forestry Ministry guidelines for handling animals, decree 87849, license A 75–05–22). Protocols have been approved by the local ethical committee “Charles Darwin” (file Ce5/2011/039). KIS knock-out mice [14] were backcrossed for 10 generations to a C57BL6/N background.

### Antibodies

As available anti-KIS antibodies were not efficient to detect endogenous KIS in western blot experiments, we raised a rat monoclonal anti-KIS antibody. A His-tagged C-terminal domain of rat KIS (aa 293–419) was expressed in bacteria, solubilized in 8 M urea, purified on Ni++column (Qiagen) followed by anion exchange on mono-Q column (Pharmacia) and renaturation by dialysis. 50 µg of the purified protein were injected intraperitoneally (i.p.) and subcutaneously (s.c.) into LOU/C rats using incomplete Freund's adjuvant supplemented with 5 nmol CpG 2006 (TIB MOLBIOL, Berlin, Germany). After a six weeks interval a final boost with 50 µg KIS and CpG 2006 was given i.p. and s.c. three days before fusion. Fusion of the myeloma cell line P3X63-Ag8.653 with the rat immune spleen cells was performed according to standard procedures. Hybridoma supernatants were tested in a solid-phase immunoassay with KIS or an irrelevant His-taged fusion protein coated to ELISA plates. Antibodies from tissue culture supernatant bound to KIS were detected with HRP conjugated mAbs against the rat IgG isotypes (TIB173 IgG2a, TIB174 IgG2b, TIB170 IgG1 all from ATCC, R-2c IgG2c homemade), thus avoiding mAbs of IgM class. HRP was visualized with ready to use TMB (1-StepTM Ultra TMB-ELISA, Thermo). MAbs that reacted specifically with KIS were further tested in western blot experiments and supernatant 3B12 (of IgG2b) was used in this study. SF1 antibodies were raised in rabbit against a N-terminal peptide (MATGANATPLDFPS). Commercial antibodies were mouse anti-U2AF^65^ (clone MC3; Sigma), mouse anti-β-actin (clone AC-15; Sigma), mouse anti-β-tubulin (clone E7, Developmental Studies Hybridoma Bank) and mouse anti-SAP155 (clone D221-3; MBL).

### Immunoblotting

Protein extracts were prepared by homogeneization of frozen tissues in TrisCl 10 mM pH 7.5, NaCl 50 mM and complete inhibitor mix (Roche Diagnostic). Nonidet P40 was then added to 0.5% final concentration and protein concentration was determined by the Biuret method (BCA kit from Pierce). After blotting, nitrocellulose sheets were stained with Ponceau red to check for homogenous loading and transfert. Specific proteins were then revealed with primary and 800 nm-IRDye-conjugated secondary antibodies (Rockland) using an Odyssey infrared imaging system (Li-Cor Biosciences). Rat monoclonal anti-KIS were detected with a secondary mouse monoclonal anti-rat IgG followed by 800 nm-IRDye-conjugated anti-mouse antibodies.

### Two-dimensional gel electrophoresis

Protein extracts were prepared from neonate brains in 10 mM TrisCl pH 7.5, 1 mM EDTA, 0.5% Nonidet P40 and complete inhibitor mix (Roche Diagnostic). 400 µg of soluble proteins were analysed for SF1 isoforms distribution on 2D PAGE as previously described [36], with a first dimension on 13 cm pH 6–11 strips using a Ettan IPG-phor 3 apparatus (GE Healthcare) and 10% acrylamide SDS-gels for the second dimension.

### RNA samples preparation

Brain were removed after decapitation of the animals and immediately frozen in liquid nitrogen and kept at −80°C until use. For RNA preparation frozen material was reduced to powder in liquid nitrogen using a mortar and pestle and 30 mg of powder was homogenized in extraction buffer and processed for total RNA preparation using Qiagen RNeasy kit. RNA quality was assessed by nanodrop and agilent Bioanalyzer.

### Microarrays analyses

RNA samples were prepared from brains of +/+ and −/− females neonate littermates (six pairs) and processed for Affymetrix Exon 1.0 ST hybridization. Raw data were processed using the Affymetrix Expression Console software suite. Normalization at the exon level was performed using the “quantile normalization”. The PLIER algorithm was used to estimate probeset signals. Expression data for each probeset was then exported into an excell spreadsheet. Probesets with a relative standard deviation (ratio of standard deviation to the mean) above 0.4 for either genotype were rejected. The fold change values for a gene (f) was arbitrarily determined from the mean fold changes for each probeset as


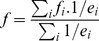
, where 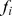 is the mean value of the fold changes for probeset i and 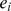 is the relative standard deviation for the fold changes for probeset i.

f is therefore the mean of the fold changes of the probesets weighted by the inverses of the relative variations.

Then we arbitrarily calculated a “confidence value” (v) for each gene expression change as 
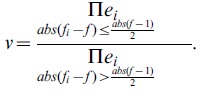
.

Intuitively, the lower “confidence value” are expected for genes with multiple probesets giving similar fold changes with low variations. This approach proved useful in detecting gene expression changes as confirmed by the Q-PCR analyses.

The complete microarray data are MIAME compliant and have been deposited in the Gene Expression Omnibus database (Accession number: GSE38097).

### Gene ontology analysis

Gene ontology analysis was performed by submitting lists of genes to the online software package of the Panther classification system server at http://www.pantherdb.org [19].

### Q-RT-PCR analyses

1 µg of total RNA was used in reverse transcription reaction using Improm II reverse transcriptase and random primers (Promega). 1/20 of each reaction was used for one Q-PCR point. Primer sequences are available upon request. Amplified products were checked for single band after agarose gel electrophoresis. Values were normalized across samples using GAPDH mRNA levels. Each real-time PCR was performed in triplicate. For pre-mRNA analyses, RNA samples were treated with RNase-free DNase (Promega) to prevent genomic DNA amplification and for each primer pairs we checked that no amplification was obtained when reverse transcriptase was omitted. For neonates, brains of 8 pairs of +/+ and −/− female littermates and 8 pairs of +/+ and −/− male littermates were used. For adults, brains of 12 pairs of +/+ and −/− 4 to 8 months old female littermates were used. The sequences of the primers used in this study are listed in Table S1.

### Neuritic extension analyses

Embryos from heterozygous mating were removed at day 14 and their cortex was dissected. After trypsin digestion 5,000 cells were seeded in 24 wells plates on glass coverslips coated with poly-L-ornithine (Sigma-Aldrich) in neurobasal medium supplemented with 2% *B*-27 and 1% penicillin/streptomycin in a 5% CO2 incubator. After four days, cells were fixed with 2% paraformaldehyde and processed for immunofluorescence staining with anti-acetylated tubulin to allow an easier automatic quantification of neurites morphology. Conventional fluorescence microscopy was performed on a Leica microscope (DM 6000) equipped with a MicroMax CCD camera (Princeton Instruments). The Metamorph software was used (Roper Scientific) for image acquisition. For each coverslip, 200 fields were acquired automatically using a motorized platform and ×20 objective. Cell morphology was analyzed using the neuronJ plugin to ImageJ [38]. Quantification was made blind to the genotype that was determined from a piece of embryo tail. At least 150 neurons were analyzed for each primary culture.

### Behavioral analysis

Groups of male animals for behavioral studies were obtained by mating heterozygous animals. For each litter all males were selected if comprising at least one −/− and one +/+ or +/− animals. Adult animals were aged from 4 to 8 months and stalled with all siblings of same gender from weaning date. As no significant difference were observed between +/+ and +/− groups, unpaired t-test analyses were performed for comparison of −/− and (+/+ and +/−) groups. In the present study, behavioral tests were performed in the following order: eyeblink and righting reflex, forepaw reaching response, circular maze activity, rotarod, hidden food test, Y maze, plus maze, open field, and either fear conditioning or prepulse inhibition tests. All experiments were conducted by an experimenter who was blind to the genotype of animals.

### Spontaneous locomotor activity in a corridor maze

Locomotor activity was measured in a circular corridor with four infrared beams placed at every 90° (Imetronic, Pessac, France) in a low-luminosity environment. Counts were incremented by consecutive interruption of two adjacent beams (i.e., mice moving through one-quarter of the circular corridor). Animal activity was monitored during 2 hours.

#### Forepaw reaching response

In this test the animal is lifted by the base of the tail to a height of approximately 15 cm and lowered to a grey bench surface within 1/2 to 1 s, decelerating as the surface was approached. Normal vision is assessed by observing if the animal extends its forelimbs toward it before reaching the surface.

#### Rotarod test

Mice were placed on a rotating 3 cm diameter drum. The drum initially was rotated at a speed of four rpm and was gradually accelerated to 40 rpm over 5 min. The amount of time the mouse remained on the accelerating rod was measured as an indicator of motor performance. This test was performed three times a day with a 30 min interval. The best score of the three trials was used for statistical analyses. Animals were tested for four consecutive days.

#### Hidden food test

The protocol was adapted from ref [39]. Animals were given small pieces of raisins in addition to their normal pellet for four days and were deprived of food for the following night. The next day, animals were placed individually in a clean box where a piece of raisin had been hidden one cm below the sawdust. The location of the food and the starting position of the mouse were the same for each animal. The latency to feed was recorded.

### Spontaneous alternation

The apparatus consisted of three identical arms and a central area with a dim illumination (2 lux) in a sound attenuated room. The activity was automatically recorded by three infrared beams in each arm for 10 minutes. An entry in the least recently visited arm was scored as an alternation. The ratio of alternation was the number of alternations over the total number of arm entries. Animals with less than eight arms entries were rejected from the statistical analysis.

### Elevated Plus maze

The elevated plus maze was used to evaluate mice emotionality. The apparatus was custom made from grey plexiglas and consisted of a central 5×5 cm area and four arms 30×5 cm. Two opposite arms were surrounded by 10 cm high walls (closed arms) the two other arms (open arms) were surrounded by 5 mm high walls to limit the aversive sensation. Arms were at an elevation of 70 cm and illuminated with 80 lux white light. Animals were placed in the central area facing an open arm and then recorded for 6 minutes with a video recording system (Viewpoint). The time spent and distance travelled in open and closed arms as well as the number of entries in open and closed arms was automatically calculated and analyzed for differences among genotypes.

### Open field

Animals were placed in a corner of a square arena (50×50 m) covered with sawdust with an homogenous 100 lux illumination at the floor level and 40 cm high white plexiglas walls, and recorded for 30 min using a video system (Viewpoint). The number of entries in a central 36×36 cm square area, the distance travelled and time spent in the central area and the total distance travelled were calculated and analyzed for differences between genotypes.

### Fear conditioning

Fear conditioning was performed using an apparatus consisting in a clear rectangular plexiglas box with a floor of parallel metallic rods for the delivery of electric current (Med Associates Inc). The first day animals were let exploring freely the new environment for two min, then a 70 dB white noise was delivered for 20 s at the end of which a concomitant 0.4 mA electric current was delivered through the metallic rods for 2 s. Animals were then let in the cage for an additional 45 s before returning to their home cage.

On day 2, animals were introduced again in the conditioning box and monitored for freezing for three minutes and then returned to their home cage. Two hours later animals were placed in an unrelated environment consisting of a circular arena the floor of which was covered with saw dust, with a cup filled with 5 ml of an odorant concentrated orange extract disposed nearby. Freezing was then recorded for three minutes without tone and for three further minutes with the tone used for conditioning. Freezing time in the novel context without tone was subtracted to the freezing time in the conditioning context and in the cued situation.

Evaluation of pain sensitivity: The reaction of animals to the electric shock was evaluated during the conditioning procedure. Reactivity was evaluated using the following notation grid: 0: no response, 1: running; 2: running and moderate jumping; 3: running and important jumping or vocalizing; 5: jumping and vocalizing.

### Startle and prepulse inhibition

Startle response and pre-pulse inhibition were assessed using two startle chambers (San Diego Instruments). The protocol used was exactly as described in [40].

### Statistical analysis

Results are expressed as means ±SEM. For qRT-PCR analyses paired student t-tests were performed. For behavioral analyses, as no significant difference between +/+ and +/− groups were observed, unpaired student t-test were used to compare −/− and (+/+ and +/−) groups. Statistical analyses were performed with Prism 3.0 software (GraphPad Software, San Diego, CA).

## Supporting Information

Table S1 **List of the primers used for Q-RT-PCR analyses.** The positions of the primers and their sequences are presented.
(XLSX)

## References

[1] Qiu Z, Ghosh A (2008) A brief history of neuronal gene expression: regulatory mechanisms and cellular consequences. Neuron 60: 449–455. 18995819 10.1016/j.neuron.2008.10.039

[2] Li Q, Lee JA, Black DL (2007) Neuronal regulation of alternative pre-mRNA splicing. Nat Rev Neurosci 8: 819–831. 17895907 10.1038/nrn2237

[3] Manceau V, Swenson M, Le Caer JP, Sobel A, Kielkopf CL, et al (2006) Major phosphorylation of SF1 on adjacent Ser-Pro motifs enhances interaction with U2AF65. FEBS J 273: 577–587. 16420481 10.1111/j.1742-4658.2005.05091.x PMC1949809

[4] Kramer A (1992) Purification of splicing factor SF1, a heat-stable protein that functions in the assembly of a presplicing complex. Mol Cell Biol 12: 4545–4552. 1406644 10.1128/mcb.12.10.4545-4552.1992 PMC360381

[5] Kramer A, Utans U (1991) Three protein factors (SF1, SF3 and U2AF) function in pre-splicing complex formation in addition to snRNPs. Embo J 10: 1503–1509. 1827409 10.1002/j.1460-2075.1991.tb07670.x PMC452814

[6] Abovich N, Rosbash M (1997) Cross-intron bridging interactions in the yeast commitment complex are conserved in mammals. Cell 89: 403–412. 9150140 10.1016/s0092-8674(00)80221-4

[7] Berglund JA, Abovich N, Rosbash M (1998) A cooperative interaction between U2AF65 and mBBP/SF1 facilitates branchpoint region recognition. Genes Dev 12: 858–867. 9512519 10.1101/gad.12.6.858 PMC316625

[8] Berglund JA, Chua K, Abovich N, Reed R, Rosbash M (1997) The splicing factor BBP interacts specifically with the pre-mRNA branchpoint sequence UACUAAC. Cell 89: 781–787. 9182766 10.1016/s0092-8674(00)80261-5

[9] Kent OA, Reayi A, Foong L, Chilibeck KA, MacMillan AM (2003) Structuring of the 3′ splice site by U2AF65. J Biol Chem 278: 50572–50577. 14506271 10.1074/jbc.M307976200

[10] Selenko P, Gregorovic G, Sprangers R, Stier G, Rhani Z, et al (2003) Structural basis for the molecular recognition between human splicing factors U2AF65 and SF1/mBBP. Mol Cell 11: 965–976. 12718882 10.1016/s1097-2765(03)00115-1

[11] Boehm M, Yoshimoto T, Crook MF, Nallamshetty S, True A, et al (2002) A growth factor-dependent nuclear kinase phosphorylates p27(Kip1) and regulates cell cycle progression. Embo J 21: 3390–3401. 12093740 10.1093/emboj/cdf343 PMC126092

[12] Maucuer A, Camonis JH, Sobel A (1995) Stathmin interaction with a putative kinase and coiled-coil-forming protein domains. Proc Natl Acad Sci U S A 92: 3100–3104. 7724523 10.1073/pnas.92.8.3100 PMC42112

[13] Caldwell BD, Darlington DN, Penzes P, Johnson RC, Eipper BA, et al (1999) The novel kinase peptidylglycine alpha-amidating monooxygenase cytosolic interactor protein 2 interacts with the cytosolic routing determinants of the peptide processing enzyme peptidylglycine alpha-amidating monooxygenase. J Biol Chem 274: 34646–34656. 10574929 10.1074/jbc.274.49.34646

[14] Langenickel TH, Olive M, Boehm M, San H, Crook MF, et al (2008) KIS protects against adverse vascular remodeling by opposing stathmin-mediated VSMC migration in mice. J Clin Invest 118: 3848–3859. 19033656 10.1172/JCI33206 PMC2582439

[15] Bieche I, Manceau V, Curmi PA, Laurendeau I, Lachkar S, et al (2003) Quantitative RT-PCR reveals a ubiquitous but preferentially neural expression of the KIS gene in rat and human. Brain Res Mol Brain Res 114: 55–64. 12782393 10.1016/s0169-328x(03)00132-3

[16] Cambray S, Pedraza N, Rafel M, Gari E, Aldea M, et al (2009) Protein kinase KIS localizes to RNA granules and enhances local translation. Mol Cell Biol 29: 726–735. 19015237 10.1128/MCB.01180-08 PMC2630681

[17] Maucuer A, Ozon S, Manceau V, Gavet O, Lawler S, et al (1997) KIS is a protein kinase with an RNA recognition motif. J Biol Chem 272: 23151–23156. 9287318 10.1074/jbc.272.37.23151

[18] Arning S, Gruter P, Bilbe G, Kramer A (1996) Mammalian splicing factor SF1 is encoded by variant cDNAs and binds to RNA. Rna 2: 794–810. 8752089 PMC1369416

[19] Thomas PD, Kejariwal A, Campbell MJ, Mi H, Diemer K, et al (2003) PANTHER: a browsable database of gene products organized by biological function, using curated protein family and subfamily classification. Nucleic Acids Res 31: 334–341. 12520017 10.1093/nar/gkg115 PMC165562

[20] Puri V, McQuillin A, Choudhury K, Datta S, Pimm J, et al (2007) Fine mapping by genetic association implicates the chromosome 1q23.3 gene UHMK1, encoding a serine/threonine protein kinase, as a novel schizophrenia susceptibility gene. Biol Psychiatry 61: 873–879. 16978587 10.1016/j.biopsych.2006.06.014

[21] Puri V, McQuillin A, Datta S, Choudhury K, Pimm J, et al. (2008) Confirmation of the genetic association between the U2AF homology motif (UHM) kinase 1 (UHMK1) gene and schizophrenia on chromosome 1q23.3. Eur J Hum Genet. 10.1038/ejhg.2008.76 18414510

[22] Powell SB, Zhou X, Geyer MA (2009) Prepulse inhibition and genetic mouse models of schizophrenia. Behav Brain Res 204: 282–294. 19397931 10.1016/j.bbr.2009.04.021 PMC2735602

[23] Valjent E, Corbille AG, Bertran-Gonzalez J, Herve D, Girault JA (2006) Inhibition of ERK pathway or protein synthesis during reexposure to drugs of abuse erases previously learned place preference. Proc Natl Acad Sci U S A 103: 2932–2937. 16473939 10.1073/pnas.0511030103 PMC1413817

[24] Kielkopf CL, Lucke S, Green MR (2004) U2AF homology motifs: protein recognition in the RRM world. Genes Dev 18: 1513–1526. 15231733 10.1101/gad.1206204 PMC2043112

[25] Manceau V, Kielkopf CL, Sobel A, Maucuer A (2008) Different requirements of the kinase and UHM domains of KIS for its nuclear localization and binding to splicing factors. J Mol Biol 381: 748–762. 18588901 10.1016/j.jmb.2008.06.026 PMC2632974

[26] Guth S, Valcarcel J (2000) Kinetic role for mammalian SF1/BBP in spliceosome assembly and function after polypyrimidine tract recognition by U2AF. J Biol Chem 275: 38059–38066. 10954700 10.1074/jbc.M001483200

[27] Tanackovic G, Kramer A (2005) Human splicing factor SF3a, but not SF1, is essential for pre-mRNA splicing in vivo. Mol Biol Cell 16: 1366–1377. 15647371 10.1091/mbc.E04-11-1034 PMC551499

[28] Zhu R, Heaney J, Nadeau JH, Ali S, Matin A (2010) Deficiency of splicing factor 1 suppresses the occurrence of testicular germ cell tumors. Cancer Res 70: 7264–7272. 20736371 10.1158/0008-5472.CAN-10-0820 PMC2940986

[29] Mazroui R, Puoti A, Kramer A (1999) Splicing factor SF1 from Drosophila and Caenorhabditis: presence of an N-terminal RS domain and requirement for viability. Rna 5: 1615–1631. 10606272 10.1017/s1355838299991872 PMC1369883

[30] Shitashige M, Satow R, Honda K, Ono M, Hirohashi S, et al (2007) Increased susceptibility of Sf1(+/−) mice to azoxymethane-induced colon tumorigenesis. Cancer Sci 98: 1862–1867. 17900258 10.1111/j.1349-7006.2007.00629.x PMC11159411

[31] Joyce CJ (2007) In silico comparative genomic analysis of GABAA receptor transcriptional regulation. BMC Genomics 8: 203. 17603907 10.1186/1471-2164-8-203 PMC1934366

[32] Steiger JL, Russek SJ (2004) GABAA receptors: building the bridge between subunit mRNAs, their promoters, and cognate transcription factors. Pharmacol Ther 101: 259–281. 15031002 10.1016/j.pharmthera.2003.12.002

[33] Alexander R, Beggs JD (2010) Cross-talk in transcription, splicing and chromatin: who makes the first call? Biochem Soc Trans 38: 1251–1256. 20863294 10.1042/BST0381251

[34] Rutz B, Seraphin B (2000) A dual role for BBP/ScSF1 in nuclear pre-mRNA retention and splicing. EMBO J 19: 1873–1886. 10775271 10.1093/emboj/19.8.1873 PMC302019

[35] Betcheva ET, Mushiroda T, Takahashi A, Kubo M, Karachanak SK, et al (2009) Case-control association study of 59 candidate genes reveals the DRD2 SNP rs6277 (C957T) as the only susceptibility factor for schizophrenia in the Bulgarian population. J Hum Genet 54: 98–107. 19158809 10.1038/jhg.2008.14

[36] Dumaine A, Maucuer A, Barbet A, Manceau V, Deshommes J, et al (2011) Genetic and molecular exploration of UHMK1 in schizophrenic patients. Psychiatr Genet 21: 315–318. 21399567 10.1097/YPG.0b013e3283458a37

[37] Bristow GC, Lane TA, Walker M, Chen L, Sei Y, et al (2009) Expression of kinase interacting with stathmin (KIS, UHMK1) in human brain and lymphoblasts: Effects of schizophrenia and genotype. Brain Res 1301: 197–206. 19747464 10.1016/j.brainres.2009.08.090 PMC2783906

[38] Meijering E, Jacob M, Sarria JC, Steiner P, Hirling H, et al (2004) Design and validation of a tool for neurite tracing and analysis in fluorescence microscopy images. Cytometry A 58: 167–176. 15057970 10.1002/cyto.a.20022

[39] Begou M, Volle J, Bertrand JB, Brun P, Job D, et al (2008) The stop null mice model for schizophrenia displays [corrected] cognitive and social deficits partly alleviated by neuroleptics. Neuroscience 157: 29–39. 18804150 10.1016/j.neuroscience.2008.07.080

[40] Spooren W, Mombereau C, Maco M, Gill R, Kemp JA, et al (2004) Pharmacological and genetic evidence indicates that combined inhibition of NR2A and NR2B subunit containing NMDA receptors is required to disrupt prepulse inhibition. Psychopharmacology (Berl) 175: 99–105. 14985927 10.1007/s00213-004-1785-y

[41] Ferguson RE, Carroll HP, Harris A, Maher ER, Selby PJ, et al (2005) Housekeeping proteins: a preliminary study illustrating some limitations as useful references in protein expression studies. Proteomics 5: 566–571. 15627964 10.1002/pmic.200400941

[42] Gozani O, Potashkin J, Reed R (1998) A potential role for U2AF-SAP 155 interactions in recruiting U2 snRNP to the branch site. Mol Cell Biol 18: 4752–4760. 9671485 10.1128/mcb.18.8.4752 PMC109061

